# Socioeconomic Deprivation and Inequalities in Mental Well-Being during the COVID-19 Pandemic among Adolescents

**DOI:** 10.3390/ijerph20136233

**Published:** 2023-06-27

**Authors:** Helena Jeriček Klanšček, Lucija Furman

**Affiliations:** National Institute of Public Health, Trubarjeva 2, 1000 Ljubljana, Slovenia; lucija.furman@nijz.si

**Keywords:** inequalities, mental well-being, adolescents, deprivation, pandemic

## Abstract

The COVID-19 pandemic highlighted the existing inequalities in education and mental health. The aim of this study was to examine socioeconomic disadvantages and mental well-being inequalities among Slovenian adolescents in October 2020. The study used nationally representative data from 3052 adolescents aged 14 and 18 (*M*_age_ = 14.4 and 18.4). The WHO-5 Well-Being Index (WHO-5) was used to measure mental well-being and risk for depression. Multinomial logistic regression was used to identify differences in the pattern of associations regarding sociodemographic characteristics and experiences during the pandemic with poor well-being and risk for depression. Our study found that adolescents from socially disadvantaged families faced poorer conditions as regards academic performance, had fewer opportunities to socialise with friends online, and were more likely to feel lonely; they reported lower levels of mental well-being and were at a higher risk for depression. The unemployment of both parents and adolescents’ perceptions of family wealth were found to be the most important predictors of depression risk. In addition, experiencing deprivation and economic hardship during the pandemic was also identified as a significant predictor. The study concludes that social and economic conditions were critical determinants of adolescents’ mental health during the pandemic and that effective intervention is needed to promote their well-being and reduce inequalities.

## 1. Introduction

The COVID-19 pandemic had a significant impact on various aspects of adolescents’ daily lives [1,2,3,4]. Adolescence is a critical developmental period and is characterised by significant changes in physical, psychological, and social functioning. The pandemic disrupted many of the important experiences that typically occur during adolescence, such as attending school, participating in extracurricular/recreational activities, and socialising with peers [5]. The restrictions imposed by the pandemic also disrupted the daily routines and structures of adolescents’ lives [6]. While the world grappled with the virus and its associated health and economic impacts, adolescents had to adjust to a new normal that was very different from what they were used to.

One way the pandemic affected adolescents’ lives was through changes in their education and school performance [7]. School closures and distance learning disrupted the traditional learning environment and created additional challenges for adolescents, such as difficulty accessing technology, connecting with classmates, maintaining focus and motivation, and coping with feelings of loneliness and social isolation [8,9]. The pandemic also resulted in the elimination of physical and social activities, which can be an important source of social support and personal development for adolescents [5]. Adolescents likely had varying levels of parental support for remote learning during the COVID-19 pandemic, depending on whether their parents had to work from home or if there were other changes in their work processes.

The COVID-19 pandemic highlighted existing inequalities in access to technology and the digital divide, which had a significant impact on educational opportunities for adolescents. The shift to distance learning has drawn attention to inequities in access to technology. Adolescents who lack access to reliable technology, high-speed Internet, and digital literacy are at a disadvantage compared to their better-off peers [8,10,11,12,13,14]. In addition, the pandemic caused economic hardship for many families, which may disproportionately affect adolescents’ health [15], mental well-being [6,16,17], relationships [18], and health behaviours [19].

All these disruptions can have a significant impact on adolescent well-being and mental health [17,20]. There is increasing evidence of mental health problems in children and adolescents during the pandemic [21,22,23]; studies describe an average doubling of clinically elevated anxiety (21%) and depression symptoms (25%) [22].

The existing literature on adults has identified mental health and mental well-being inequalities during the COVID-19 pandemic. Females, younger people, and people of low socioeconomic status (SES) are more likely to report mental health-related problems [24,25,26]. However, fewer studies focused only on mental well-being inequalities among adolescents during the COVID-19 pandemic. Studies found that females [4,27,28,29,30,31] and older adolescents reported lower mental health or well-being more often [21,32,33,34]. Some studies examined specific socioeconomic indicators related to adolescent mental health during the COVID-19 pandemic, but inequalities were not their main focus. For example, Ravens-Sieberer et al. [4] examined changes in health-related quality of life (HRQoL) and mental health in children and adolescents (*n* = 1923) aged 7 to 17 years and their parents in order to identify the associated risk and resource factors during the pandemic. Socially disadvantaged children and children of mentally burdened parents were at particular risk of impaired mental health. A Chinese study of 175,416 adolescents [21] showed that being female and/or of an older age were associated with higher levels of depression, anxiety, and lower levels of post-traumatic growth (PTG). Symptoms related to COVID-19, excessive attention to epidemic information, living in urban or severe epidemic areas, and conflicts with parents during home quarantine were significant factors affecting depression, anxiety, and PTG. Poor family economic status was a significant risk factor for depression and PTG. In the study conducted by Folch et al. [35], 57% of the participants (*n* = 303, 14–18 years old) reported poor overall mental health, with females reporting worse mental health than males. Participants reporting a decrease in their family’s income were more likely to report poor overall well-being. The preliminary results of a study from Luxemburg show that females and young people with low socioeconomic status coped less well with the overall pandemic situation [6].

Despite the widespread negative consequences of the pandemic, it is possible that some individuals experienced positive personal changes as an adaptive response to this global crisis (e.g., greater appreciation of life, stronger bonds with others) [36]. PTG can occur after a potentially traumatic event, leading to enhanced personal strength, openness to new possibilities, deeper relationships with others, greater appreciation of life, and spiritual development [37,38]. During the pandemic, the majority of adolescents quickly adapted to the new life situations of insecurity and dealing with unfavourable and adverse conditions by seeking out the conditions of the new normalcy and finding alternative solutions in daily life [39]. The COVID-19 pandemic had a significant impact on adolescents’ lives, affecting their education, social relationships, and mental health. Understanding these impacts is critical to developing effective interventions to support adolescent well-being. Although studies suggest that some social and economic conditions are important determinants of adolescent mental health during the pandemic, further research is needed. There is a lack of empirical evidence focusing exclusively on inequalities in mental well-being and related factors during the pandemic. The aim of this study is to examine and extend the evidence for digital inequalities and inequalities in mental well-being and associated factors among 14- and 18-year-olds in Slovenia during the pandemic.

## 2. Materials and Methods

### 2.1. Participants and Procedure

We used nationally representative data from 9th-grade primary school (PS) students and 4th-year secondary school (SS) students. The sample consisted of 3052 adolescents: 1854 primary school students (51.4% male, *M*_age_  =  14.4  ±  0.5 years) and 1198 secondary school students (46.7% male, *M*_age_ =  18.4  ±  0.5 years).

The survey was based on a two-stage stratified sampling frame. The sampling unit was the class. We collected data on a number of 9th-grade classes in all primary schools (941 class units in 489 schools) and on a number of 4th-year classes in all secondary schools (557 class units in 152 schools) from the Ministry of Education’s database. In the first stage, we sampled primary and secondary schools; and in the second stage, secondary schools were further sampled according to programme (gymnasium, technical, or other professional education). The sample size consisted of 112 classes in primary schools and 67 classes in secondary schools, with an average class size of 22.63 students. The principals of the schools selected in the sample were asked to participate in the survey. Schools obtained written informed consent from the parents of participating students prior to their participation in the study. The students completed the online survey on school computers as well as on tablets and smartphones. The final response rate was 91%.

The data for this study a connected to the Health Behaviour in School-aged Children (HBSC) study, which is an international cross-sectional survey on adolescent health. During the COVID-19 pandemic in October of 2020, schools were asked to participate in an unscheduled round of data collection with the HBSC survey questionnaire, which included questions and scales on sociodemographic characteristics, the health-related behaviours and habits of adolescents, their lifestyles, and their experiences regarding the COVID-19 pandemic. The study was approved by the Slovenian Ethics Committee (No. 0120-469/2020/8).

### 2.2. Instruments

#### 2.2.1. WHO-5—Mental Well-Being

The WHO-5 Well-Being Index measures mental well-being in the previous two weeks and it can also be used as a screening tool for depression. It covers five positively worded items related to positive mood, vitality, and general interests. The raw score is calculated by summing the responses to the five items. To obtain a standardised percentage score ranging from 0 to 100, the raw score is multiplied by 4 [40]. Having good well-being is considered to be the case when the percentage score falls into a range from 51 to 100. A percentage score between 29 and 50 represents poor well-being; the risk for depression is considered to exist when the percentage score is 28 or below [41]. The internal consistency measured by Cronbach’s alpha in this study was α = 0.88.

#### 2.2.2. Sociodemographic Measures

The sociodemographic and socioeconomic data included self-reported information on sex, grade, type of family, parental employment, and perceived family wealth. All sociodemographic measures were defined as categorical variables. Sex included two categories: male and female. Grade included two categories: 9th grade in PS and 4th grade in SS. Type of family was also classified into two groups: nuclear and non-nuclear family (single-parent, reconstructed, etc.). Parental employment was classified into three groups: both parents employed, one parent unemployed, or both unemployed. How the adolescents perceived their family’s wealth was grouped into three categories: average, below average, and above average.

#### 2.2.3. COVID-19 Related Measures

COVID-19-related measures can be divided into two time-related parts. The first one refers to the adolescents’ experience during the pandemic, when the adolescents were at school but with various preventive measures in place. The second refers to adolescents’ experience during the country’s lockdown.

Experiences during the pandemic (report in October 2020)

- Deprivation and economic hardship

Deprivation and economic hardship were assessed by means of eight items on a three-point scale (yes, no, I do not know/I do not remember) [42]. Examples of items: “One or both of my parents lost their job”, “There was less money at home”. The items were summed to obtain the degree of deprivation and economic hardship that adolescents experienced during the pandemic. The internal consistency measured by Cronbach’s alpha in this study was α = 0.69.

- Post-traumatic personal growth

Items adapted from the Revised Post-traumatic Growth Inventory for Children (PTGI-C-R) [43] were used to assess the degree of adolescents’ post-traumatic personal growth. The original PTGI-C-R includes two open-ended items and ten items assessing five PTG domains (new possibilities, relating to others, personal strength, appreciation of life, and spiritual change) on a four-point scale (no change, a little change, some change, a lot of change, do not know).

We used eight items (without two items related to spiritual change and open-ended items). Example items are as follows: “I learned how nice and helpful some people can be”, “I know what is important to me better than I used to”. The internal consistency measured by Cronbach’s alpha in this study was α = 0.92.

Experiences during the COVID-19 lockdown (report in October 2020 regarding spring 2020)

- Digital access and connectedness

We measured digital access and connectedness during the COVID-19 lockdown by six items on a five-point scale (never, rarely, sometimes, often, very often) [42]. The items were related to having a digital device that the adolescent could use to access the internet for schoolwork, having access to online alternatives to schoolwork and teaching, having support and help with schoolwork and learning, having access to online alternatives for meeting up with classmates and friends, having one’s own space at home for schoolwork, and receiving enough information regarding the state of the country.

- Feelings of loneliness and isolation

We assessed adolescents’ feelings of loneliness and isolation during the COVID-19 lockdown using a three-item five-point scale (never, rarely, sometimes, often, very often) questionnaire [42]. The items were as follows: “How often did you feel lonely during the COVID-19 pandemic?”, “How often did you feel that you were part of a group of friends during the COVID-19 pandemic?” and “Were there people you could talk to about things that are important to you during the COVID-19 pandemic?”.

### 2.3. Statistical Analysis

Population weights on gender and secondary school programmes were used in the sample to achieve the representativeness of the school population of Slovenia. We grouped participants into categories as regards mental well-being based on their WHO-5 percentage score (i.e., good well-being, poor well-being, risk of depression).

First, we performed descriptive statistics and assessed the association between sociodemographic characteristics (i.e., sex, grade, type of family, parental employment, and perceived family wealth) and categories of mental well-being during the pandemic, using a bivariate χ^2^ analysis. The same analysis was used to examine adolescents’ experiences during the lockdown (i.e., digital access and connectedness, feelings of loneliness and isolation) by sociodemographics characteristics.

In the next step, we used multinomial logistic regression in order to identify differences in the pattern of associations of sociodemographic characteristics and experiences during the pandemic (i.e., post-traumatic personal growth, deprivation, and economic hardship) (predictors), with poor well-being and risk for depression (outcomes), respectively, relative to good well-being (the reference group). The odds ratios and their 95% confidence intervals were calculated. To check for multicollinearity between the independent variables, a multilinear regression model was first run. The variance inflation factor values—which indicate whether a predictor has a strong linear relationship with the other predictor—for all variables were <10, and tolerance > 0.1, indicating no multicollinearity between them. Data analysis was carried out using IBM SPSS Statistics, Version 25. The significance level was set at 0.05.

## 3. Results

### 3.1. The Sociodemographic Characteristics of the Study Participants

In total, 3052 adolescents participated in the study: 1854 primary school students (51.4% male, *M*_age_  =  14.4  ±  0.5 years) and 1198 secondary school students (46.7% male, *M*_age_  =  18.4  ±  0.5 years). The majority (80.8%) of the adolescents lived in a nuclear family, with the remaining 19.2% in non-nuclear families (single-parent, reconstructed, etc.). As many as 86.5% of the adolescents reported that both parents were employed, and 12.5% that only one was employed. Only 1% of the adolescents had both parents unemployed. A total of 61.8% of the adolescents perceived their family’s wealth as above average, 33.3% of the adolescents as average, and 4.9% of the adolescents as below average.

### 3.2. The Pandemic: Mental Health Inequalities

Table 1 summarises the cross tabulation of the mental health categories with various sociodemographic characteristics. The data show that 55.5% of the adolescents had good well-being, while 25.9% had poor well-being and 18.8% were at risk for depression. Females were at greater risk for depression than males, and older adolescents were more likely to have symptoms of depression than younger ones. Adolescents from non-nuclear families and from families where both parents were unemployed had a higher risk for depression. In addition, adolescents who rated their family’s wealth as average or below average were more likely to experience poor well-being or be at risk for depression. The observed associations are small in strength, except in the case of gender.

As mentioned above, the pandemic caused economic hardship for many families. During the pandemic, 45.5% of the adolescents experienced deprivation and economic hardship and rated their family’s economic situation as worse. The adolescents who perceived their family wealth to be below average (χ^2^ = 108.530; *p* < 0.0001), who were from non-nuclear families (χ^2^ = 24.139; *p* < 0.0001), and with at least one unemployed parent (χ^2^ = 13.571; *p* = 0.0002) were more likely to report that their family’s economic situation was worse than before the pandemic.

Finally, 14.4% of the adolescents experienced post-traumatic personal growth during the pandemic and saw it as an opportunity for personal growth. The adolescents who rated their family’s wealth as above average were more likely to experience post-traumatic personal growth (χ^2^ = 22.337; *p* < 0.0001).

#### Multinomial Logistic Regression Representing the Predictors of Poor Well-Being and Risk for Depression

Table 2 shows the results of the multinomial logistic regression model used to examine the predictors associated with poor well-being and risk for depression compared to good mental health.

Regarding the fitted model information, the chi-square test of the full model versus the intercept-only model yielded a significant result, χ^2^ (18) = 348.730, *p* < 0.001, indicating that the predictors as a group distinguished satisfactorily between the WHO-5 categories. The analyses revealed a good fit of the model fit to the data; χ^2^ (4464) = 4328.769, *p* = 0.925 (using the deviance criterion); the pseudo R2 Nagelkerke was 0.141.

The multinomial logistic model correctly classified 58.3% of all adolescents with a focus on the mental health categories based on the above predictor variables. We evaluated its usefulness by comparing the percentage of correctly classified cases with the proportion by chance rate (PCC). PCC was 41.3%. A model is useful if the total classification rate is higher than the chance criterion (i.e., PCC multiplied by 1.25) (see [44]), which, in our case, was 51.6%. Results confirm that the model is useful.

The results suggest that being a female is significantly associated with both poor well-being and risk for depression, with females twice as likely as males to experience poor well-being and three times as likely to experience depression. Subjective assessment of one’s family’s wealth is also a significant predictor of both poor well-being and risk for depression. The adolescents who rated their family’s wealth as below average or average were more likely to experience poor well-being and have a higher risk for depression than those who rated it as above average.

Post-traumatic personal growth was negatively associated with risk for depression, implying that it decreases the odds of risk for depression and can have a protective role. Deprivation and economic hardship are also significant predictors of both poor well-being and the risk for depression, with adolescents who have experienced hardship more likely to have poor well-being and be at risk for depression.

The effects of family type and parental employment were small and not significant for poor well-being, but significant for risk for depression, especially for when both parents were unemployed, which is the most important predictor of the risk for depression. The adolescents for whom both parents were unemployed had almost four times the risk for depression than the adolescents for whom both parents were employed. Adolescents with both parents unemployed and with low perceived family wealth are the most vulnerable group and have a higher risk for depressive symptoms.

### 3.3. COVID-19 Lockdown: Digital Inequalities and Feelings of Loneliness and Isolation

In line with our research aim, we wanted to examine adolescents’ digital access and connectedness, as well as feelings of loneliness and isolation during the COVID-19 lockdown, and determine if there were significant differences across various sociodemographic characteristics.

The results showed that a significant percentage of the adolescents experienced difficulty accessing resources for education and communication during the pandemic lockdown. As many as 10.2% of the adolescents had difficulty (i.e., reported never, rarely, or occasionally having access) with access to electronic devices, 10.1% with access to online tools for school, and 10.8% with environments for communicating with friends. A quarter (26.6%) of the adolescents received help and support with schoolwork occasionally or less frequently, and 15.5% of the adolescents rarely had a dedicated space at home for schoolwork. One-fifth of the adolescents (21.4%) never, rarely, or occasionally received enough information about the situation in the country during the pandemic.

In addition, nearly one fifth (19.6%) of the adolescents often or very often felt lonely during the lockdown and never or rarely felt like they were part of a group of friends (19.9%). As many as16.6% of the adolescents never or rarely had anyone to talk to.

Univariate associations of the previously mentioned variables by sociodemographic/economic indicators can be found in Table A1. We highlight only the socioeconomic indicators of the family (i.e., family type, the employment of parents, perceived family wealth). The percentage of adolescents who received help and support with schoolwork occasionally or less often was higher among the adolescents with below-average perceived family wealth (χ^2^ = 30.289; *p* < 0.0001) than among the adolescents with an above-average assessment thereof. The same applies to whether they have their own space for schoolwork at home (χ^2^ = 36.944; *p* < 0.0001). The percentage of adolescents who rarely had a place at home to be alone while doing schoolwork was also higher among the adolescents from non-nuclear families (χ^2^ = 9.663; *p* = 0.002) and among those with at least one parent unemployed (χ^2^ = 18.895; *p* < 0.0001). The adolescents from non-nuclear families also faced challenges related to accessing resources for education and communication at a higher rate than the adolescents from nuclear families (χ^2^ = 6.92; *p* = 0.009 and χ^2^ = 6.26; *p* = 0.012, respectively).

Feelings of loneliness and not belonging to a group of friends were experienced by a higher percentage of adolescents with lower-than-average perceived family wealth (χ^2^ = 44.259; *p* < 0.0001 and χ^2^ = 13.880; *p* = 0.001, respectively) and among adolescents from non-nuclear families (χ^2^ = 16.621; *p* < 0.0001 and χ^2^ = 37.037; *p* < 0.0001, respectively). The percentage of adolescents who never or rarely had someone to talk to was also higher among adolescents from non-nuclear families (χ^2^ = 8.240; *p* = 0.004).

## 4. Discussion

Our study found that adolescents aged 14 to 18 from socially disadvantaged families in the large sample of this study had poorer conditions for schoolwork, fewer opportunities for social contact with friends, and more frequent feelings of loneliness during the first lockdown of the pandemic in spring 2020 compared to more privileged adolescents in Slovenia. Adolescents from socioeconomically disadvantaged families also reported lower levels of mental well-being and were at a greater risk for depression (as of October 2020). We identified several predictors of poor well-being and the risk for depression. The most important predictor of depression risk was found to be the unemployment of both parents and adolescents’ perception of family wealth as being below average. In addition, deprivation and economic hardship during the pandemic contributed significantly to the risk of depression among adolescents.

However, our findings are consistent with previous studies showing that almost all EU countries faced digital gaps, leading to inequalities in educational opportunities caused by the pandemic, especially due to the lack of computer equipment among students, among other factors [8,10,11,12,13,14].

A study from Cyprus [45] reported that 10% of students (very similar to our study) did not have access to a reliable digital device, while one in ten did not have access to reliable software/communication tools. In addition, 19.8% did not have access to a reliable internet connection, 51.2% owned their own computer or laptop, and almost a third (27%) owned a tablet. Rzanova et al. [46] also observed digital inequalities and found that 7.6% of survey respondents reported using a mobile phone, and 27.4% of students owned a laptop or tablet but shared it with another family member during the school day. Pigaiani et al. [47] reported that two-thirds of Italian students had a computer or tablet at home, 74% had internet, and 59% had both internet and a computer or tablet. These findings, like our study, indicate that there are digital inequalities among young people.

Compared to our results, Lessard and Puhl [48] found that more American (39%) than Slovenian youth (25%) receive no help or support with schoolwork.

In a Norwegian study, Kaiser et al. [49] analysed Norwegian adolescents’ perceptions of and satisfaction with information related to the COVID-19 pandemic [49]. Nearly half of the adolescents (49%) expressed satisfaction or high satisfaction with the information they received about COVID-19. On the other hand, 12% expressed dissatisfaction or high dissatisfaction, while 39% reported being neutral or neither satisfied nor dissatisfied. Comparing Slovenian and Norwegian adolescents, it can be seen that Norwegian adolescents were more satisfied with the information they received from the authorities about COVID-19. This could be due to better communication from the authorities or a higher level of trust in the government; level of trust in the Slovenian government was low during the pandemic [50].

Consistent with previous findings, we confirmed the inequalities of educational opportunities and inequalities in support and loneliness [34,51]. Adolescents from less affluent families and those from non-nuclear families faced particular challenges in accessing school resources and support during the pandemic. They were more likely to experience loneliness and feelings of not belonging during the pandemic. A significantly higher percentage of them reported feeling lonely and not belonging to a group of friends, compared to those from more affluent families. Additionally, a considerable proportion of adolescents reported never or seldom having someone to talk to.

In Slovenia, efforts were made to provide devices and internet access to students in need, but these initiatives were limited and could not reach all students. Overall, the pandemic highlighted the importance of addressing the digital gap and providing equal access to technology for all students, especially during times of remote learning. A multifaceted approach is needed to address this inequality. This includes providing devices and internet access, developing digital literacy skills, and promoting equitable policies that support equal access to technology.

Our study was one of the few studies on the inequality of mental well-being among adolescents during the pandemic. More than half (55.3%) of the adolescents (14 and 18 years) rated their mental well-being as good, and 18.8% reported risk for depression after the first lockdown and summer holidays and before the second lockdown in October 2020 in Slovenia. This is consistent with a Lithuanian study from 2022, where more than half (58%) of the 11–19-year olds reported good well-being and 18.8% reported a risk for depression [41]. It should be noted that in the 2018 HBSC study in Slovenia, 65.0% of the adolescents (11, 13, 15, and 17 years old) reported good well-being and 13.4% reported depression risk [52].

As expected, our study suggests that several sociodemographic factors, such as being female, coming from a non-nuclear family where both parents are unemployed, and lower perceived family wealth, are associated with lower mental well-being. This is consistent with previous studies showing that the risk for mental health problems during the pandemic was higher for females than for males. Similarly, results from an Italian study [53] examining adolescents’ perceived mental well-being (WHO-5) found that females scored worse than males on the WHO-5 questionnaire, indicating poorer mental health. The same was found in studies from Lithuania [41], Austria [54,55] and the Netherlands [56] (which all found lower WHO-5 scores in females compared to males). In addition, Jusienė et al. [41] and Holm-Hadulla et al. [57] found that females were more likely to report emotional and behavioural problems lasting longer than one year and had more COVID-19 anxiety, suggesting poorer mental health than males, and further supporting our findings. It has been suggested that the explanation for the poorer mental health in female adolescents may be related to COVID-19 restrictions. Magson et al. [58] reported that the pandemic constraints (e.g., online schooling, social distancing) affected adolescent females’ ability to rely on their social network for emotional support during stressful times, which could lead to a deterioration in their mental well-being.

In addition, Dunton et al. [59], Ellis and Zarbatany [60], and Kang et al. [61] observed that females were more likely to engage in sedentary behaviours, use more social media, spend more time in front of screens, and sleep more than males during confinement—all behaviours which have been linked to an increase in mental health problems. In addition, Halldorsdottir et al. [62] reported that females were more likely than males to perceive that the pandemic had a negative impact on their daily lives and school performance and affected their mental well-being. Furthermore, in addition to social factors, biological factors can also influence females’ mental well-being. Martel [63] suggests that hormonal influences during puberty increase sensitivity to interpersonal stressors in adolescent females and that they are more likely than males to engage in social behaviours (discussing problems in dyadic relationships) under stress that exacerbate depressive symptoms and consequently worsen their mental well-being [64].

Our results show that adolescents from non-nuclear families (e.g., with a single parent) were associated with a higher risk for depression than adolescents from nuclear families. Consistent with our findings, Wolf and Schmitz [65] and Hafstad et al. [66] confirmed that children and adolescents growing up with a single parent were particularly at risk for developing mental health problems during the pandemic. Prior to the pandemic, it was emphasised that the source of the observed differences may be complex and may include macro and micro explanations (e.g., socioeconomic background, insecurity and participation, the cultural and social preferences of families with two parents, family resources, climate, etc.) [67].

The examination of various (inequality) indicators revealed that unemployed parents and low perceived material wealth (along with gender) were the strongest predictors of the risk for depression. The association between adverse financial events such as unemployment and financial deprivation, parental stress, and their negative impact on adolescents’ emotional well-being is well known from before the pandemic and has been described in the family stress model [68]. The interaction between parental financial stress and adolescents’ psychological well-being was also analysed by Low and Mounts [69], who found that there was a significant relationship between greater financial stress and poorer adolescents’ mental well-being (e.g., internalizing behaviour and loneliness) due to parental psychological stress. Frasquilho et al. [70] argued that parental distress during unemployment is associated with changes in adolescents’ emotional problems and parent–adolescent relationships, which may negatively affect adolescents’ psychological well-being. In addition, Lawson et al. [71] reported that parents who lost their jobs due to the pandemic were almost five times more likely to psychologically abuse their children, which further worsened their psychological well-being and increased their risk for developing anxiety and depression later in life [72]. Finally, Neppl et al. [73] showed that conflict between parents and adolescents resulted in fewer young people seeking parental social support in times of crisis. It is known that the pandemic period limited adolescents’ socialization with their friends [74]; therefore, when there was less parental support due to parental conflict [73], some adolescents received very little social support, which can lead to feelings of loneliness [69], increased use of digital media [75], and poorer psychological well-being [76]. Furthermore, in line with our findings, a study from Greece [77] found that parental unemployment was associated with an increase in family conflict and a deterioration in parental psychological well-being, which, in turn, negatively affected adolescents’ well-being.

In addition, several studies reported that self-assessed financial status was associated with adolescent mental health during the pandemic. A study from Germany [78] found strong evidence of an uneven distribution of illness complexity among young people in different socioeconomic groups, with more young people with complex chronic conditions living in families with low socioeconomic status. Young people from low socioeconomic backgrounds were repeatedly found to be more likely to have mental health problems. Similarly, a study from China [74] reported that low socioeconomic status and lower parental education levels were associated with a higher incidence of mental health problems among young people. They confirmed that the association between parental education and mental health problems was significant among adolescents in the provinces with the lowest GDP per capita, suggesting that the poorest adolescents living in the most deprived areas were the most affected by mental health problems during the pandemic.

A small percentage of adolescents experienced post-traumatic personal growth (PTG) during the pandemic and saw it as an opportunity for personal growth. Interestingly, adolescents who rated their family’s wealth as above average were more likely to experience this type of growth. Similar to our findings, Zhou et al. [79] observed a positive association between a family’s economic status and adolescents’ post-traumatic stress growth in a study of Chinese adolescents (PTG). Higher family financial status was associated with higher levels of post-traumatic stress growth in adolescents. In addition, a study from China [80] showed that higher subjective socioeconomic status (SES) predicted higher levels of PTG in university students. Finally, a positive relationship between higher SES and higher PTG was also confirmed in a study by Augustine [81]. As Zhou et al. [79] found, the development of post-traumatic stress was strongly influenced by adolescents’ relationships with their parents.

Despite the various contributions this work makes to our understanding of how children were affected by the pandemic, some limitations need to be discussed. First, the study used a cross-sectional design, which limits the ability to make causal inferences. Future research should use longitudinal designs to examine the causal relationships between socioeconomic status, stressful event-related experiences, and changes in daily routines and mental well-being among adolescents. Second, the study relied on self-reported measures, which may be subject to social desirability bias. The use of different methods to collect data on the same phenomenon is desirable. Third, although a wide range of instruments has been used, others which are more precise and not considered here, such as a family affluence scale, should also be included in future studies. Finally, future research should examine the impact of the pandemic on specific subgroups of adolescents, such as those with pre-existing mental health conditions, to better understand their unique needs during the pandemic.

## 5. Conclusions

Adolescents are vulnerable to mental health inequalities. Factors such as being female, being older, coming from a non-nuclear family, both parents being unemployed, and perceived lower family wealth are associated with lower mental well-being. Parental unemployment is the most important predictor of depression risk among adolescents, and adolescents with unemployed parents are the most vulnerable group to inequalities regarding mental well-being. On a positive note, post-traumatic personal growth is found to be a factor protecting against poor well-being and depression risk. Thus, interventions to promote personal growth and build resilience may be helpful for adolescents who are vulnerable to mental health inequalities. Overall, addressing socioeconomic disadvantages and promoting social support may be important strategies for reducing mental health inequalities among adolescents.

## Figures and Tables

**Table 1:** Mental well-being categories by study variables.

		Risk for Depression	Poor Well-Being	Good Well-Being	χ^2^
Total		18.8%	25.9%	55.3%	
Gender	Male	12.3%	21.6%	66.1%	146.56, *p* < 0.001 Cramer’s V = 0.22
	Female	25.1%	30.0%	44.9%	
Grade	9th (PS)	17.4%	24.7%	57.9%	12.89, *p* = 0.002 Cramer’s V = 0.07
	4th (SS)	21.1%	27.5%	51.4%	
Type of family	Nuclear	17.0%	25.3%	57.7%	29.61, *p* < 0.001 Cramer’s V = 0.10
	Non-nuclear	25.5%	28.2%	46.3%	
Parental employment	Both employed	17.6%	25.4%	57.1%	16.74, *p* = 0.002 Cramer’s V = 0.06
	One unemployed	21.1%	28.3%	50.6%	
	Both unemployed	42.3%	26.9%	30.8%	
Perceived family wealth	Above average	14.8%	23.4%	61.8%	107.85, *p* < 0.001 Cramer’s V = 0.13
	Average	23.7%	30.1%	46.2%	
	Below average	37.5%	29.2%	33.8%	
Deprivation and economic hardship	Yes	24.6%	28.9%	46.5%	88.62, *p* < 0.001 Cramer’s V = 0.17
	No	14.0%	23.3%	62.7%	
Post-traumatic personal growth	Yes	11.6%	21.7%	66.7%	29.12, *p* < 0.001 Cramer’s V = 0.10
	No	20.1%	26.8%	53.1%	

χ^2^: Chi-square test. *p*: *p*-value. Cramer’s V: measure of association.

**Table 2:** Parameter estimates of the multinomial logistic regression.

		Poor Well-Being ^a^				Risk for Depression ^a^		
	B (S.E)	*p*	Exp(B)	95% CI for Exp(B)	B (S.E)	*p*	Exp(B)	95% CI for Exp(B)
Intercept	−1.649 (0.388)	0.001			−2.343 (0.453)	0.001		
Females ^1^	0.724 (0.096)	0.001	2.062	1.710–2.487	1.161 (0.116)	0.001	3.194	2.544–4.009
Age	0.016 (0.024)	0.501	1.016	0.970–1.065	0.022 (0.028)	0.427	1.022	0.968–1.049
Perceived family wealth ^2^								
Below average	0.571 (0.275)	0.038	1.770	1.032–3.034	0.955 (0.281)	0.001	2.598	1.499–4.504
Average	0.448 (0.103)	0.001	1.566	1.280–1.916	0.560 (0.119)	0.001	1.751	1.387–2.211
Type of family ^3^								
Non-nuclear	0.229 (0.132)	0.082	1.257	0.971–1.626	0.413 (0.145)	0.004	1.512	1.137–2.011
Parental employment ^4^								
Both unemployed	0.647 (0.548)	0.238	1.911	0.652–5.596	1.326 (0.537)	0.014	3.767	1.315–10.789
One unemployed	0.043 (0.145)	0.766	1.044	0.786–1.387	0.066 (0.165)	0.689	1.068	0.774–1.475
Post-traumatic personal growth	−0.016 (0.008)	0.040	0.984	0.970–0.999	−0.054 (0.010)	0.001	0.948	0.930–0.965
Deprivation and economic hardship	0.213 (0.041)	0.001	1.237	1.142–1.341	0.329 (0.044)	0.001	1.390	1.276–1.514

Reference category: ^a^—good well-being. Groups compared: ^1^—males; ^2^—above average; ^3^—nuclear; ^4^—both employed. *p*: *p*-value. Exp(B): odds ratio. CI: confidence interval.

## Data Availability

The data presented in this study are available on request from the National Institute of Public health: https://nijz.si/podatki/podatkovne-zbirke-in-raziskave/raziskava-z-zdravjem-povezano-vedenje-v-solskem-obdobju-hbsc-2018/ (accessed on 27 October 2022).

## Appendix A

**Table A1:** Digital access and connectedness and feelings of loneliness and isolation during the COVID-19 lockdown by sociodemographic characteristics.

		Never, Rarely, or Occasionally …						Often or Very Often …	Never or Seldom …	
		Have Access to Electronic Devices	Have Access to Online Tools for School	Have Access to Environments to Communicate with Friends	Received Help and Support with Schoolwork	Had Their Own Space at Home for Schoolwork	Received Enough Information during the Pandemic	Felt Lonely	Had Feeling of Being a Part of a Group of Friends	Had People to Talk to
Total		10.2%	10.1%	10.8%	26.6%	15.5%	21.4%	19.6%	19.9%	16.6%
Gender	Males	14.6%	15.0%	16.4%	29.8%	18.3%	23.8%	14.4%	20.7%	19.7%
	Females	5.9%	5.3%	5.4%	23.5%	12.8%	19.0%	24.6%	19.2%	13.7%
	χ^2^	60.83; *p* < 0.001	76.53; *p* < 0.001	90.96; *p* < 0.001	15.18; *p* < 0.001	16.77; *p* < 0.001	10.31; *p* < 0.001	48.74; *p* < 0.001	1.08; *p* = 0.299	19.12; *p* < 0.001
Grade	9th (PS)	12.2%	12.0%	13.6%	25.7%	17.2%	20.3%	16.4%	21.2%	19.5%
	4th (SS)	7.3%	7.1%	6.5%	28.0%	13.0%	23.0%	24.5%	17.9%	12.4%
	χ^2^	18.27; *p* < 0.001	18.42; *p* < 0.001	36.98; *p* < 0.001	2.06; *p* = 0.152	9.56; *p* = 0.002	3.39; *p* = 0.066	29.55; *p* < 0.001	4.87; *p* = 0.027	25.59; *p* < 0.001
Type of family	Nuclear	9.0%	9.1%	9.8%	25.7%	14.2%	19.8%	18.3%	17.4%	15.3%
	Non-nuclear	14.0%	12.8%	13.4%	29.5%	19.4%	27.3%	25.9%	28.7%	20.3%
	χ^2^	13.22; *p* < 0.001	6.92; *p* = 0.009	6.26; *p* = 0.012	3.32; *p* = 0.069	9.66; *p* = 0.002	15.29; *p* < 0.001	16.62; *p* < 0.001	37.04; *p* < 0.001	8.24; *p* = 0.004
Parental employment	Both employed	9.4%	9.6%	10.3%	26.6%	14.0%	20.4%	19.0%	18.6%	15.9%
	One unemployed	10.9%	9.5%	11.7%	25.7%	18.3%	23.1%	19.8%	21.3%	17.9%
	Both unemployed	7.4%	7.4%	3.7%	29.6%	40.7%	22.2%	35.7%	28.6%	29.6%
	χ^2^	0.92; *p* = 0.630	0.15; *p* = 0.930	1.91; *p* = 0.384	0.21; *p* < 0.899	18.89; *p* < 0.001	1.37; *p* = 0.505	5.01; *p* = 0.082	3.12; *p* = 0.210	3.32; *p* = 0.069
Perceived family wealth	Above average	10.1%	10.4%	11.2%	23.3%	13.5%	18.2%	16.7%	18.3%	16.9%
	Average	9.9%	9.0%	9.8%	31.3%	16.8%	25.6%	22.5%	21.3%	15.2%
	Below average	12.0%	12.8%	12.2%	38.3%	32.1%	32.7%	37.9%	30.4%	22.1%
	χ^2^	0.56; *p* = 0.755	2.59; *p* = 0.273	1.65; *p* = 0.438	30.29; *p* < 0.001	36.94; *p* < 0.001	32.42; *p* < 0.001	44.26; *p* < 0.001	13.88; *p* = 0.001	4.55; *p* = 0.103

χ^2^: Chi-square test. *p*: *p*-value.
